# Revealing the Distribution of Aggregation-Induced Emission Nanoparticles via Dual-Modality Imaging with Fluorescence and Mass Spectrometry

**DOI:** 10.34133/2021/9784053

**Published:** 2021-06-19

**Authors:** Liucheng Mao, Yuming Jiang, Hui Ouyang, Yulin Feng, Ruoxin Li, Xiaoyong Zhang, Zongxiu Nie, Yen Wei

**Affiliations:** ^1^The Key Laboratory of Bioorganic Phosphorus Chemistry & Chemical Biology (Ministry of Education), Department of Chemistry, Tsinghua University, Beijing 100084, China; ^2^Beijing National Laboratory for Molecular Sciences, Key Laboratory of Analytical Chemistry for Living Biosystems, Institute of Chemistry, Chinese Academy of Sciences, Beijing 100190, China; ^3^State Key Laboratory of Innovative Drug and Efficient Energy-Saving Pharmaceutical Equipment, Jiangxi University of Traditional Chinese Medicine, Nanchang 330006, China; ^4^Department of Chemistry, Nanchang University, 999 Xuefu Avenue, Nanchang 330031, China

## Abstract

Aggregation-induced emission nanoparticles (AIE NPs) are widely used in the biomedical field. However, understanding the biological process of AIE NPs via fluorescence imaging is challenging because of the strong background and poor penetration depth. Herein, we present a novel dual-modality imaging strategy that combines fluorescence imaging and label-free laser desorption/ionization mass spectrometry imaging (LDI MSI) to map and quantify the biodistribution of AIE NPs (TPAFN-F127 NPs) by monitoring the intrinsic photoluminescence and mass spectrometry signal of the AIE molecule. We discovered that TPAFN-F127 NPs were predominantly distributed in the liver and spleen, and most gradually excreted from the body after 5 days. The accumulation and retention of TPAFN-F127 NPs in tumor sites were also confirmed in a tumor-bearing mouse model. As a proof of concept, the suborgan distribution of TPAFN-F127 NPs in the spleen was visualized by LDI MSI, and the results revealed that TPAFN-F127 NPs were mainly distributed in the red pulp of the spleen with extremely high concentrations within the marginal zone. The *in vivo* toxicity test demonstrated that TPAFN-F127 NPs are nontoxic for a long-term exposure. This dual-modality imaging strategy provides some insights into the fine distribution of AIE NPs and might also be extended to other polymeric NPs to evaluate their distribution and drug release behaviors *in vivo*.

## 1. Introduction

Luminescent materials have attracted attention for their utility in chem-/biosensors [1–3], photoelectric devices [4–6], and biomedical diagnosis or therapy [7–10]. As one of the most promising luminescent materials, aggregation-induced emission luminogens (AIEgens) are nearly nonluminescent in solution while highly luminescent in the aggregate or solid state [11]. The fascinating photoluminescence (PL) behavior and intrinsic hydrophobicity of AIEgens are particularly beneficial for the fabrication of AIEgen-based nanomaterials [12]. Typically, polymer-encapsulated AIEgen-containing nanoparticles (AIE NPs) with features such as desirable size, stable brightness, high resistance to photobleaching, and excellent biocompatibility have been used as fluorescent contrast reagents for *in vitro* or *in vivo* bioimaging and disease diagnostic reagents [13–19]. Recently, through rational design of AIEgens, AIE NPs have been endowed with multifunctionality, including photodynamic or photothermal therapeutic ability, making them suitable for theranostic applications [20–28]. As the biomedical applications of AIE NPs become more widespread, increasing concerns have been raised about their behavior in biological systems [29]. However, fluorescence imaging inevitably suffers from limitations, such as photobleaching, strong background, and poor penetration depth [30, 31]. Determining how to depict the fine biodistribution of AIE NPs at both the microscopic and macroscopic levels remains a big challenge. Therefore, developing a complementary analysis technology to assist fluorescence imaging for mapping and quantifying the biodistribution of AIE NPs is of great significance.

Matrix-assisted laser desorption/ionization mass spectrometry imaging (MALDI MSI) has become an accurate analysis tool for the detection and quantification of targeted compounds (e.g., nucleic acids, proteins, polysaccharides, drugs, and metabolic products) in tissue sections and for mapping their spatial distribution at the suborgan or single-cell level [32–37]. Recently, a new laser desorption/ionization mass spectrometry imaging (LDI MSI) technique that does not require a matrix has received significant attention for *in vivo* analysis and mapping nanomaterials (e.g., carbon nanomaterials, gold nanoparticles, and MoS_2_ nanosheets) based on their intrinsic fingerprint signal [38–40]. This direct and label-free method avoids the potential diffusion of target compounds and interference from biomolecules. Encouraged by these studies, we hypothesized that the proposed LDI MSI method could be employed to identify the suborgan or tissue distribution of AIE NPs by detecting the molecular weight of AIEgens, as AIE nanoparticles consist of AIEgens in the core and biocompatible matrices as shells. It is noteworthy that the combination of LDI MSI and fluorescence imaging methods can enhance both the reliability and accuracy of determination. More importantly, LDI MSI could localize the materials (even their modifications) with high resolution without any background interference. Although previous studies have reported the dual/multimodel imaging applications of AIEgen-based materials, the signals originate from different components (e.g., AIEgens and gadolinium) [41, 42]. To the best of our knowledge, the application of AIEgens for fluorescence/mass spectrometry dual-model imaging that relies only on their intrinsic properties has not been reported thus far.

**Scheme 1:**
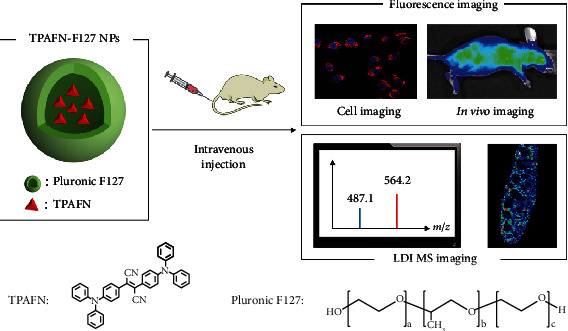
Schematic of TPAFN-F127 NPs for fluorescence imaging and LDI MSI.

In the present work, a novel dual-modality imaging technique that combines fluorescence imaging and LDI MSI by monitoring the intrinsic photoluminescence and mass spectrometry signal of an AIEgen (named as TPAFN) is reported. Pluronic F127 was chosen as the polymer matrix to improve the water dispersibility and biocompatibility of TPAFN-F127 NPs (Scheme 1). The obtained TPAFN-F127 NPs possessed a narrow size distribution, regular spherical structure, high photostability, and good chemical stability. After evaluating their biocompatibility, fluorescence imaging was conducted. An LDI MSI approach was further employed to study the localization, distribution, and quantification of AIE nanoparticles in tissues. To the best of our knowledge, this is the first study on the biodistribution of AIE NPs by LDI MSI. We hope this research will provide a deeper insight into the biological process of AIE NPs *in vivo*.

## 2. Results and Discussion

### 2.1. Preparation and Characterization of TPAFN and TPAFN-F127 NPs

**Figure 1:**
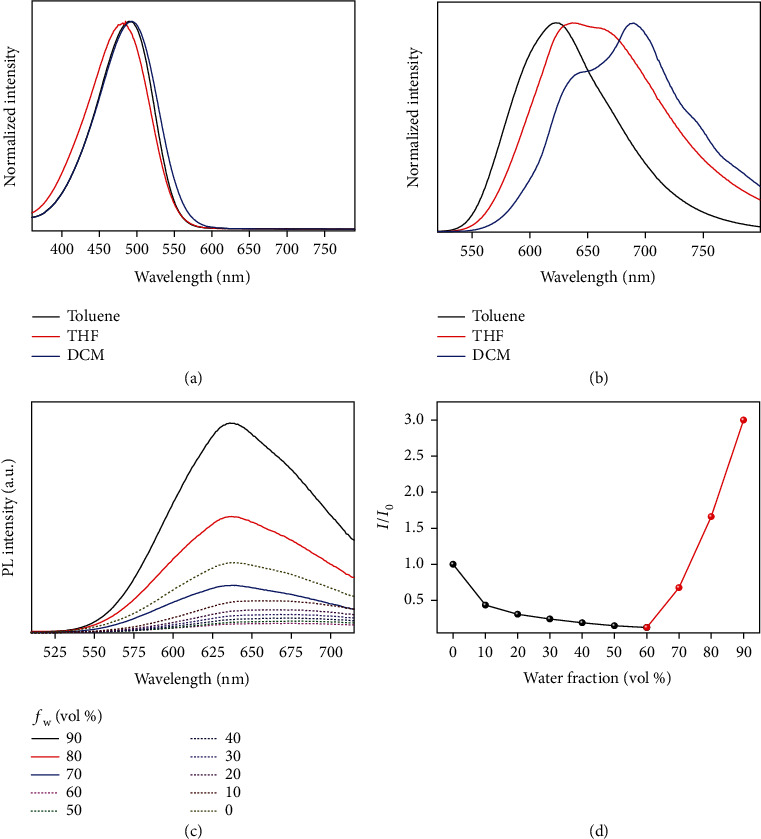
Photophysical properties of TPAFN. (a) Normalized UV-vis spectra of TPAFN in toluene, THF, and DCM. (b) Normalized PL spectra of TPAFN in toluene (Ex = 488 nm), THF (480 nm), and DCM (492 nm). (c) PL spectra of TPAFN in an aqueous THF solution with different water fractions (*f*_w_), *λ*_ex_ = 480 nm. (d) Variation in the PL intensity of TPAFN in an aqueous THF solution with different water fractions. *I*_0_ denotes the PL intensity of TPAFN in pure THF.

The intermediate product (FN-2Br) and target AIEgen (TPAFN) were synthesized according to the previously reported methods (Scheme S1) [43]. All synthetic products were verified by ^1^H/^13^C NMR spectroscopy and mass spectrometry. The specific spectroscopic data of various products are listed in Materials and Methods, and the spectra are shown in Figures S1–S6. The optimal absorption peaks of TPAFN in toluene, tetrahydrofuran (THF), and dichloromethane (DCM) were located at 488, 480, and 492 nm, respectively (Figure 1(a)). It is worth mentioning that the maximum emission of TPAFN shifted from 622 nm (toluene) to 689 nm (DCM) with an increase in the solvent polarity, which is indicative of the typical twisted intramolecular charge transfer (TICT) (Figure 1(b)). The fluorescence spectra of TPAFN in a THF/water solution were obtained, which is a common method for studying AIE property (Figure 1(c)). The emission of TPAFN in pure THF was relatively weak, and the PL intensity gradually declined, while the emission wavelength underwent a bathochromic shift before the water fraction (*f*_w_) reached 60%, demonstrating the TICT state [44]. However, the PL intensity was dramatically enhanced at high water ratios (*f*_w_ > 60%) (Figure 1(d)). These results indicate that TPAFN possesses both TICT and AIE properties.

**Figure 2:**
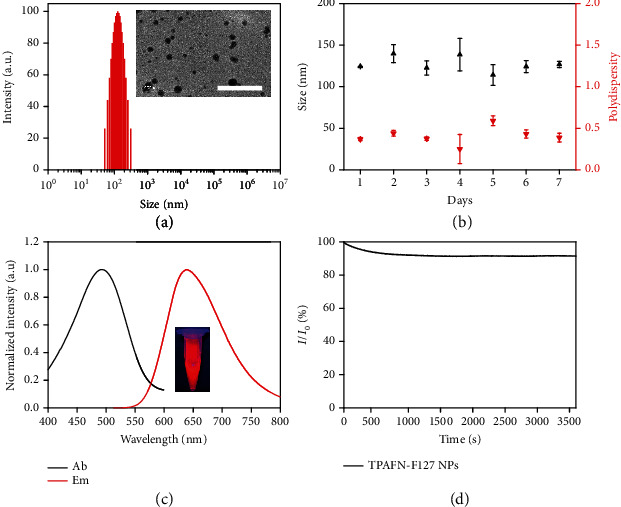
(a) Size distribution of TPAFN-F127 NPs in aqueous solution. Inset: TEM image of TPAFN-F127 NPs; scale bar: 1 *μ*m. (b) Size stability of TPAFN-F127 NPs after a 7-day storage. (c) UV-vis absorption (Ab) and emission (Em) spectra of TPAFN-F127 NPs in water. Inset: PL image of TPAFN-F127 NPs acquired under 365 nm UV illumination at room temperature. (d) Photostability evaluation of TPAFN-F127 NPs in water under irradiation (493 nm) for 1 h.

The unique AIE characteristic of TPAFN is beneficial for the fabrication of AIE nanoparticles. Pluronic F127 was employed to encapsulate TPAFN via a thin-film hydration method (Scheme S2). The average size of the TPAFN-F127 NPs was 126 nm (PDI = 0.352) (Figure 2(a)). Furthermore, TEM images indicate that the obtained TPAFN-F127 NPs possessed a spheroidal morphology and smooth surface (Figure S7). No evident changes on the hydrodynamic diameters were observed after TPAFN-F127 NPs were stored for 7 d (Figure 2(b)). The photophysical properties of TPAFN-F127 NPs were also investigated. The optimal absorption and maximum emission peaks appeared at 493 nm and 638 nm, respectively (Figure 2(c)). More importantly, good photostability of TPAFN-F127 NPs was demonstrated in water (Figure 2(d)). The fluorescence intensity was barely affected when TPAFN-F127 NPs were mixed with various pH buffers ranging from 1 to 13 (Figure S8). These conclusions suggest that TPAFN-F127 NPs have excellent potential for biological imaging.

### 2.2. Cytotoxicity and Biocompatibility of TPAFN-F127 NPs

**Figure 3:**
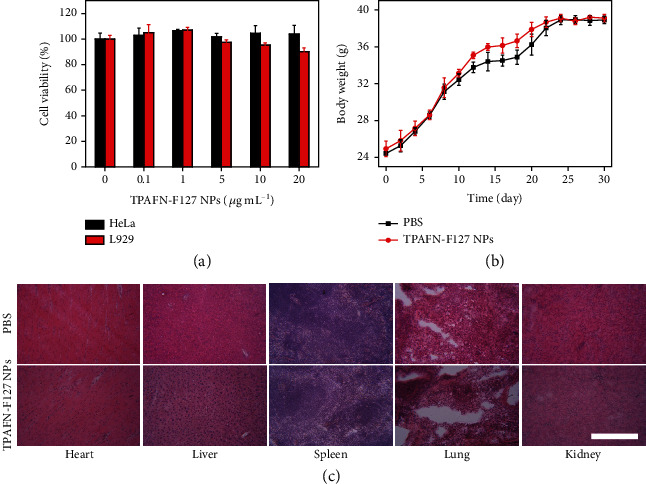
(a) Cell viability of L929 cells and HeLa cells incubated with TPAFN-F127 NPs at different concentrations for 24 h. (b) Body weight variation of mice (*n* = 5) treated with 250 *μ*L PBS and TPAFN-F127 NPs (250 *μ*L, 20 mg/kg). (c) Representative H*&*E-stained images of main organ slices collected from various groups after a 30-day treatment. Scale bar: 100 *μ*m.

The cytotoxicity of TPAFN-F127 NPs was evaluated using L929 cells and HeLa cells through the CCK-8 assay. Negligible cytotoxicity was observed even at TPAFN-F127 NP concentrations up to 20 *μ*g mL^−1^, suggesting that the NPs were biocompatible with L929 cells and HeLa cells (Figure 3(a)). Moreover, the long-term potential toxicity of TPAFN-F127 NPs was evaluated in Kunming mice. Compared to the control groups, no significant body weight variation was found in the TPAFN-F127 NPs (250 *μ*L, 20 mg/kg) (Figure 3(b)). Meanwhile, hematoxylin and eosin- (H*&*E-) stained images of the main organs (heart, liver, spleen, lung, and kidney) revealed no evidence of inflammatory lesions in both control groups and experimental groups at day 30 of posttreatment (Figure 3(c)). We further performed blood biochemical analysis to assess the hematological biocompatibility of TPAFN-F127 NPs in mice after 7 days of treatment. As shown in Table S1, a slight statistical difference was observed in all hematological parameters, even though the dose of TPAFN-F127 NPs up to 20 mg/kg demonstrated no significant inflammation. In general, the above results substantiate the biocompatibility of TPAFN-F127 NPs at an appropriate dose for biomedical applications.

### 2.3. Fluorescence Imaging of TPAFN-F127 NPs

**Figure 4:**
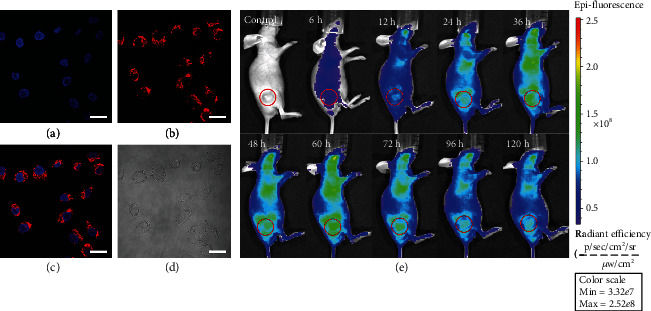
CLSM imaging of (a–d) HeLa cells incubated with TPAFN-F127 NPs for 3 h. The concentration of TPAFN-F127 NPs is 5 *μ*g mL^−1^. Scale bar: 20 *μ*m. (e) *In vivo* fluorescence imaging of HeLa tumor-bearing mice after intravenous injection with TPAFN-F127 NPs (200 *μ*L, 10 mg/kg). The tumor region is circled in red.

The *in vitro* cellular uptake of TPAFN-F127 NPs was studied using confocal laser scanning microscopy (CLSM). After HeLa and L929 cells were similarly treated with TPAFN-F127 NPs (5 *μ*g mL^−1^) for 3 h, the cells were stained with Hoechst 33258. As indicated by the CLSM images in Figures 4(a)–4(d) and Figure S9, the homogeneous red fluorescence was located in the cytoplasm and the blue region in the nucleus, suggesting that TPAFN-F127 NPs were efficiently accumulated and distributed in the cytoplasm of both HeLa cells and L929 cells. These results demonstrate that TPAFN-F127 NPs are a good candidate for biological imaging.

Noninvasive *in vivo* fluorescence imaging was also conducted using HeLa tumor-bearing mice to assess the biodistribution and tumor accumulation of TPAFN-F127 NPs. *In vivo* fluorescence imaging and tumor accumulation of TPAFN-F127 NPs in HeLa tumor-bearing mice over time are shown in Figure 4(e). The PL intensity in the tumor region becomes brighter before 60 h postinjection, indicating that TPAFN-F127 NPs accumulated in the tumor through the enhanced permeability and retention (EPR) effect (Figure S10) [45, 46]. It is important to note that the PL signals in the tumor region remained detectable even after 120 h postinjection, demonstrating that TPAFN-F127 NPs can track tumors over a long period of time. *Ex vivo* fluorescence imaging revealed much stronger fluorescence in the liver and tumor, suggesting that TPAFN-F127 NPs possess tumor-targeting efficiency (Figure S11 and S12).The existence of TPAFN-F127 NPs in the liver and spleen is due to the critical role of the reticuloendothelial system organs in the uptake and excretion of exogenous nanoparticles [47]. Furthermore, *ex vivo* fluorescence imaging of tissue slices was performed using fluorescence microscopy. As shown in Figure S13, no fluorescence was observed for the control group, but bright red spots were observed in the tissues of mice after intravenous injection with TPAFN-F127 NPs, which could be ascribed to the TPAFN-F127 NP signal.

### 2.4. Biodistribution of TPAFN-F127 NPs in Mice Revealed by LDI MSI

**Figure 5:**
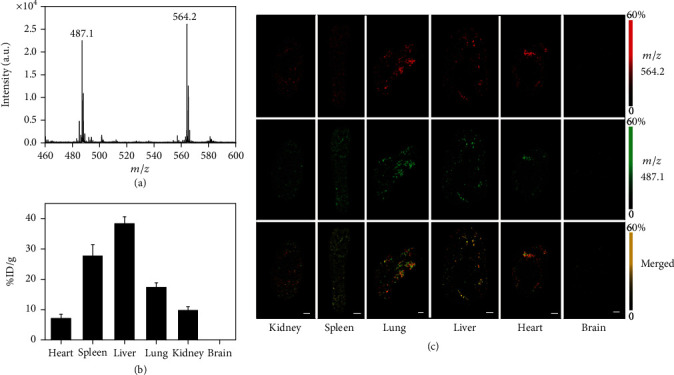
(a) Typical LDI MS spectrum of TPAFN-F127 NPs. (b) Quantification of TPAFN-F127 NPs in various mouse organs detected by LDI MS. %ID/g denotes the percentage of injected dose per gram of tissue. (c) LDI MS images reveal the biodistribution of TPAFN-F127 NPs in different organs. Scale bars: 1 mm.

According to the intrinsic properties (multiple aromatic ring structures for good laser adsorption), TPAFN can be detected using LDI MS without any matrix, which greatly eliminates diffusion and improves the accuracy of positioning. Figure S6 presents the typical LDI MS spectrum of TPAFN without any matrix assistance; the peaks at *m*/*z* 564.2 and 487.1 correspond to the ions [TPAFN]^−^ and [TPAFN-C_6_H_5_]^−^, respectively. Moreover, TPAFN-F127 NPs exhibited the same LDI MS signal as pure TPAFN, which indicated that Pluronic F127 matrix encapsulation did not change the intrinsic molecular weight of TPAFN, and TPAFN-F127 NPs could also be determined by LDI MS (Figure 5(a)). No ion signal was detectable in any tissue of blank mice by LDI MS without matrix assistance (Figure S14). By integrating the results from the control group with high physicochemical stability of TPAFN-F127 NPs, the expected *m*/*z* 564.2 and 487.1 in tissues (kidney, spleen, lung, liver, heart, and brain) of TPAFN-F127 NP-injected mice could be ascribed to the ion signal originating from injected TPAFN-F127 NPs (Figure S15). Further LDI MSI experiments were performed to quantify the deposition amount and analyze the biodistribution of TPAFN-F127 NPs in various organs. As shown in Figures 5(b) and 5(c), TPAFN-F127 NPs were clearly observed via active strong-to-weak sequences of various organs. The variation of ion signal intensity in various tissue slices was consistent with *ex vivo* fluorescence imaging, indicating that the reciprocation was realized. More importantly, it is evident that the integration of LDI MSI and fluorescence imaging enhanced both the reliability and accuracy of determination. TPAFN-F127 NPs in various organs were quantified using the calibration curve acquired from each tissue spiked with TPAFN-F127 NPs (see details in Figure S16). The results showed that TPAFN-F127 NPs mainly accumulated in the liver compared to other organs. In particular, no detectable fingerprint peaks of TPAFN-F127 NPs were found in the brain, which can be attributed to the low efficiency of TPAFN-F127 NPs across the blood-brain barrier. This result is consistent with those of fluorescence imaging.

### 2.5. Suborgan LDI MSI of TPAFN-F127 NPs

**Figure 6:**
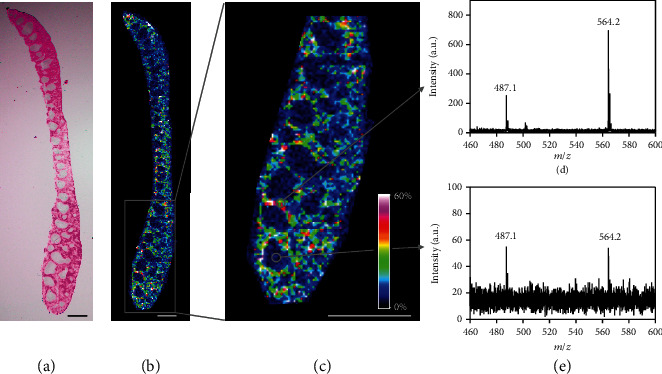
Suborgan distribution of TPAFN-F127 NPs in spleen tissue of a mouse. (a) Optical image of a spleen tissue slice. (b) Heat map to reveal the distribution (*m*/*z* 564.2) of TPAFN-F127 NPs in the spleen tissue slice. (c) Enlarged image of (b) indicates the distribution of TPAFN-F127 NPs in distinct histological regions. Scale bars: 5 mm. (d) Representative LDI MS of red pulp region. (e) Representative LDI MS of white pulp region.

Owing to the limitations of strong background and poor penetration depth, it is very difficult for fluorescence technology to image the distribution of fluorescent nanoparticles in finer tissue structures. As a complementary technique, LDI MSI is capable of revealing the chemical information and spatial distributions of target analytes within tissues. The suborgan distribution of TPAFN-F127 NPs in the spleen was characterized as a sample because the splenic tissue possesses distinguishable histological regions, including white pulp, red pulp, and a marginal zone (Figure S17) [48]. It is clear that the red pulp accumulated with a large number of TPAFN-F127 NPs, which could be because the spleen is responsible for blood filtration and clearance (Figure 6(b)) [48]. However, the deposition of TPAFN-F127 NPs in the white pulp region was much lower and even undetectable (Figure 6(e)). Furthermore, the marginal zone, the region between lymphoid white pulp and nonlymphoid red pulp, captures particulate antigens from circulation and presents antigens to lymphocytes of the spleen. Not surprisingly, a high ion intensity was found in these regions, and the quantitative results also support this conclusion (Figure 6(d) and Figure S18). This fine suborgan distribution of TPAFN-F127 NPs was first studied by LDI MSI, which provides insight into the interaction between AIE NPs and living systems.

## 3. Conclusion

In summary, we prepared AIEgen-containing luminescent nanoparticles (TPAFN-F127 NPs) with superior physicochemical properties and excellent biocompatibility, making them preferable for biological imaging. Fluorescence imaging verified that TPAFN-F127 NPs are promising as a fluorescent probe in cell imaging and tumor targeting by the EPR effect. The distribution and quantification of TPAFN-F127 NPs were investigated by LDI MSI, which demonstrated that TPAFN-F127 NPs were mainly distributed in the liver, spleen, and lung. Detailed suborgan analysis of the spleen revealed that TPAFN-F127 NPs were heavily deposited more in the marginal zone and red pulp. Therefore, dual-modality imaging technology that combines fluorescence imaging with LDI MSI could be a good candidate for studying and assessing AIEgen-based nanoparticles before clinical biomedical applications. It is worth noting that this combination method has further potential implications, such as interaction analysis (e.g., modification and oxidation) between AIE nanoparticles and endogenous molecules, depending on the comprehensive molecular analysis capability by mass spectrometry.

## 4. Materials and Methods

### 4.1. Synthesis of 2,3-Bis[4-(diphenylamino)phenyl]fumaronitrile (TPAFN)

Diphenylamine (338 mg, 2.0 mmol), FN-2Br (194 mg, 0.5 mmol), Pd(OAc)_2_ (11 mg, 0.05 mmol), and Cs_2_CO_3_ (1.2 g, 3.5 mmol) were dissolved in 30 mL toluene, and then, tri-*tert*-butylphosphine (30 mg, 0.15 mmol) was added under an N_2_ atmosphere. The reaction mixture was stirred at 110°C and monitored by thin-layer chromatography (TLC). After reaction completion, the mixture was extracted with ethyl acetate. Finally, the crude product was purified by column chromatography (petroleum ether/ethyl acetate: 10/1) to yield TPAFN: ^1^H NMR (400 MHz, d_6_-DMSO): 7.71–7.62 (*m*, *J* = 7.68 Hz, 4H), 7.42–7.37 (*m*, *J* = 7.39 Hz, 8H), 7.21–7.14 (*m*, *J* = 7.18 Hz, 12H), 6.97–6.94 (*m*, *J* = 6.95 Hz, 4H); ^13^C NMR (101 MHz, d_6_-DMSO): 150.55, 146.73, 146.43, 129.92, 129.74, 126.02, 124.80, 124.61, 121.02, and 120.42; HRMS (MALDI-TOF, *m*/*z*): calculated for C_40_H_28_N_4_ = 564.231; found = 564.150 [M]^−^.

### 4.2. Preparation of TPAFN-F127 NPs

The TPAFN-F127 NPs were prepared using the thin-film hydration method. In brief, Pluronic F127 (20 mg) and TPAFN (2 mg) were dissolved in 2 mL of THF under continuous sonication for 30 min. THF was then evaporated on a rotary evaporator at 40°C. Finally, 2 mL of deionized water was added and the mixture was sonicated for 5 min. After purification by a 0.2 *μ*m filter, the obtained TPAFN-F127 NPs were stored at 4°C. The concentration of TPAFN-F127 NPs was calculated using a UV/VIS/NIR spectrophotometer at 493 nm.

## Data Availability

The authors declare that all relevant data are included in this article and its supplementary information file. The remaining data are available from the corresponding authors upon request.

## Supplementary Materials

Supplementary Materials Scheme S1: the synthetic route of AIE molecule (TPAFN). Scheme S2: the preparation of TPAFN-F127 NPs via a thin-film hydration method. Figure S1: 1H NMR spectrum of FN-2Br. Figure S2: 13C NMR spectrum of FN-2Br. Figure S3: MALDI-TOF MS spectrum of FN-2Br. Figure S4: 1H NMR spectrum of TPAFN. Figure S5: 13C NMR spectrum of TPAFN. Figure S6: MALDI-TOF MS spectrum of TPAFN. Figure S7: TEM image of TPAFN-F127 NPs. Figure S8: the variation of fluorescent intensity of TPAFN-F127 NPs incubated with different mediums with various pH buffers. Table S1: changes of hematology parameters of mice induced by TPAFN-F127 NPs (*n* = 8). Figure S9: CLSM imaging of L929 cells after incubation with TPAFN-F127 NPs for 3 h at 37°C. The concentration of TPAFN-F127 NPs is 5 *μ*g mL^−1^. Scale bar: 20 *μ*m. Figure S10: variation in the intensity at different time points after tail-vein injection of TPAFN-F127 NPs. *I*_0_ refers to the intensity of the tumor region in the control group. Figure S11: ex vivo fluorescence imaging of tumor and major organs after 24 h postintravenous injection of TPAFN-F127 NPs (200 *μ*L, 10 mg/kg). Figure S12: histogram of the PL intensity of the organs (heart, liver, spleen, lung, and kidney), brain, and tumor. Figure S13: representative ex vivo fluorescence imaging of various types of tissue slices from mice treated with TPAFN-F127 NPs (200 *μ*L, 10 mg/kg) after 24 h postinjection; scale bar: 200 *μ*m. Figure S14: representative LDI mass spectra of normal mouse tissue slice of the liver, spleen, lung, kidney, heart, and brain in negative ion mode. Figure S15: representative LDI mass spectra of TPAFN-F127 NP-injected normal mouse tissue slice of the liver, spleen, lung, kidney, heart, and brain in negative ion mode. Figure S16: standard calibration curves for TPAFN-F127 NPs in various organs including the liver, heart, lung, kidney, and spleen. Figure S17: photograph of splenic tissues. Figure S18: quantitative results of TPAFN-F127 NPs in the white pulp, red pulp, and marginal zones in the spleen tissue section.
