# Supplementary material for: Revealing the Distribution of Aggregation-Induced Emission Nanoparticles via Dual-Modality Imaging with Fluorescence and Mass Spectrometry
Source: Research (Wash D C). 2021 Jun 19;2021:9784053. doi: 10.34133/2021/9784053 (PMC8237597; doi:10.34133/2021/9784053)
Supplement: Supplementary Materials — Scheme S1: the synthetic route of AIE molecule (TPAFN). Scheme S2: the preparation of TPAFN-F127 NPs via a thin-film hydration method. Figure S1: 1H NMR spectrum of FN-2Br. Figure S2: 13C NMR spectrum of FN-2Br. Figure S3: MALDI-TOF MS spectrum of FN-2Br. Figure S4: 1H NMR spectrum of TPAFN. Figure S5: 13C NMR spectrum of TPAFN. Figure S6: MALDI-TOF MS spectrum of TPAFN. Figure S7: TEM image of TPAFN-F127 NPs. Figure S8: the variation of fluorescent intensity of TPAFN-F127 NPs incubated with different mediums with various pH buffers. Table S1: changes of hematology parameters of mice induced by TPAFN-F127 NPs (n = 8). Figure S9: CLSM imaging of L929 cells after incubation with TPAFN-F127 NPs for 3 h at 37°C. The concentration of TPAFN-F127 NPs is 5 μg mL−1. Scale bar: 20 μm. Figure S10: variation in the intensity at different time points after tail-vein injection of TPAFN-F127 NPs. I0 refers to the intensity of the tumor region in the control group. Figure S11: ex vivo fluorescence imaging of tumor and major organs after 24 h postintravenous injection of TPAFN-F127 NPs (200 μL, 10 mg/kg). Figure S12: histogram of the PL intensity of the organs (heart, liver, spleen, lung, and kidney), brain, and tumor. Figure S13: representative ex vivo fluorescence imaging of various types of tissue slices from mice treated with TPAFN-F127 NPs (200 μL, 10 mg/kg) after 24 h postinjection; scale bar: 200 μm. Figure S14: representative LDI mass spectra of normal mouse tissue slice of the liver, spleen, lung, kidney, heart, and brain in negative ion mode. Figure S15: representative LDI mass spectra of TPAFN-F127 NP-injected normal mouse tissue slice of the liver, spleen, lung, kidney, heart, and brain in negative ion mode. Figure S16: standard calibration curves for TPAFN-F127 NPs in various organs including the liver, heart, lung, kidney, and spleen. Figure S17: photograph of splenic tissues. Figure S18: quantitative results of TPAFN-F127 NPs in the white pulp, red pulp, and marginal [file 9784053.f1.docx]

Revealing the Distribution of Aggregation-Induced Emission Nanoparticles via Dual-modality Imaging with Fluorescence and Mass Spectrometry

Liucheng Mao^1^, Yuming Jiang^2^, Hui Ouyang^3^, Yulin Feng^3^, Ruoxin Li^1^, Xiaoyong Zhang^4^, Zongxiu Nie^2^, Yen Wei^1^

^1^ The Key Laboratory of Bioorganic Phosphorus Chemistry & Chemical Biology (Ministry of Education), Department of Chemistry, Tsinghua University, Beijing 100084, China

^2^ Beijing National Laboratory for Molecular Sciences, Key Laboratory of Analytical Chemistry for Living Biosystems, Institute of Chemistry, Chinese Academy of Sciences, Beijing 100190, China

^3^ State Key Laboratory of Innovative Drug and Efficient Energy-Saving Pharmaceutical Equipment, Jiangxi University of Traditional Chinese Medicine, Nanchang 330006, China

^4^ Department of Chemistry, Nanchang University, 999 Xuefu Avenue, Nanchang 330031, China

**1. Materials and Methods**

**1.1 Materials and Characterization**

Sodium methoxide, 4-bromophenylacetonitrile, diphenylamine, palladium (II) acetate (Pd(OAc)_2_), and tri-*tert*-butylphosphine were purchased from Aladdin. Iodine and Pluronic F127 were purchased from Sigma-Aldrich. All other reagents and solvents were purchased from commercial suppliers and used without further purification unless otherwise noted.

^1^H NMR and ^13^C NMR spectra were recorded on a JEOL 400 MHz spectrometer. The high-resolution mass spectra (HRMS) were measured using an Ultraflextreme MALDI-TOF/TOF mass spectrometer (Bruker Daltonics (Billerica, MA)). The size distribution of TPAFN-F127 NPs was measured by a Brookhaven ZetaPlus zeta-potential analyzer. UV-vis absorption spectra were recorded using UV/VIS/NIR spectrometer (Perkin Elmer, Lambda 750). The emission spectra were recorded using a Shimadzu RF-6000 spectrofluorometer. The morphology of TPAFN-F127 NPs was observed by Transmission electron microscopy (TEM) (HT7700, Hitachi, Japan). The cell imaging was collected using a confocal laser scanning microscope (CLSM, Zeiss 710 3-channel, Germany).

**1.2 Synthesis of bis(4-bromophenyl)fumaronitrile (FN-2Br)**

Sodium methoxide (2.3 g, 43 mmol) was dissolved in methanol (15 mL) at -78°C, then the solution was added dropwise to a cooled (-78°C) ethyl ether solution of 4-bromophenylacetonitrile (3.9 g, 20 mmol) and iodine (5.1 g, 20 mmol). After stirring for 3 h at -78°C and another 4 h at 0ºC, HCl (aq) (3%, v/v, 60 mL) was added to quench the reaction. The obtained precipitates were obtained through filtration and washed with water (3×100 mL), Na_2_S_2_O_5_ (aq) (5%, v/v, 3 × 50 mL), and water (3 × 100 mL), respectively. The product was collected as pale yellow solids. ^1^H NMR (400 MHz, CDCl_3_): 7.73–7.66 (m, 8H); ^13^C NMR (101 MHz, CDCl_3_): 132.8, 130.7, 130.2, 126.9, 124.7, 116.2; HRMS (MALDI-TOF, m/z): calculated for C_16_H_8_Br_2_N_2_ = 387.902; found = 387.732 [M]^−^.

**1.3 Cell culture**

L929 cells and HeLa cells were cultured in a humidified atmosphere at 37°C with a 5% CO_2_ atmosphere. L929 cells were cultured in 1640 medium, and HeLa cells were cultured in Dulbecco’s Modified Eagle Medium (DMEM, Gibco), supplemented with 10% fetal bovine serum (FBS), 100 IU mL^–1^ penicillin, and 100 µg mL^−1^ streptomycin.

**1.4 Cell viability evaluation of TPAFN-F127 NPs**

The cytotoxicity of TPAFN-F127 NPs was evaluated by a standard cell counting kit-8 (CCK-8) assay. Briefly, L929 cells or HeLa cells were seeded in 96-well microplates at a density of 5 × 10^3^ cells mL^–1^ in 100 μL of cell culture medium. After overnight culture, cells were incubated with different doses of TPAFN-F127 NPs for 24 h. Then cells were washed with phosphate-buffered saline (PBS) three times. 10 μL of CCK-8 solution and 100 μL of cell culture medium were added to each well and incubated for another 2 h at 37^−1^. Finally, plates were analyzed using a microplate reader (VictorШ, PerkinElmer). Measurements of formazan dye absorbance were carried out at 450 nm, with the reference wavelength at 620 nm. The values were proportional to the number of live cells. The percent reduction of CCK-8 dye was compared to controls (cells not exposed to TPAFN-F127 NPs), which represented 100% CCK-8 reduction. Three replicate wells were used per microplate, and the experiment was operated three times. Cell survival was expressed as absorbance relative to that of untreated controls. Results are presented as mean ± standard deviation (SD).

**1.5 Cell imaging of TPAFN-F127 NPs**

The cell uptake behavior of TPAFN-F127 NPs was investigated by a CLSM using L929 cells and HeLa cells. The excitation wavelength was set as 488 nm. Cells were cultured in a glass-bottom dish with a density of 1 × 10^5^ cells per dish. On the day of treatment, TPAFN-F127 NPs with a suitable concentration of 5 μg mL^–1^ were incubated with cells for 3 h at 37°C. Afterward, the cells were washed three times with PBS and then fixed with 4% paraformaldehyde for 10 min at room temperature for cell imaging.

**1.6 Animal care and ethics statement**

Animal experiments were performed with male Kunming mice (25-30 g, Experimental Animal Center of the Academy of Military Medical Sciences) and female BALB/c nude mice (5-6 weeks, Vital River Laboratory Animal Technology, 401). The animal experiments were conducted according to the NIH Guide for the Care and Use of Laboratory Animals (National Institutes of Health Publication, No. 3040-2, revised 1999, Bethesda, MD) and were approved by the Animal Care and Use Committee of the Chinese Academy of Sciences.

**1.7 *In vivo* toxicity studies**

PBS (250 μL) and TPAFN-F127 NPs (250 μL, 20 mg/kg) were injected into the male Kunming mice via the tail vein, n = 3 per group. The weight of these mice was recorded every day. After 36 d post-injection, the mice were sacrificed and the major organs (heart, liver, spleen, lung, kidneys, and brain) were excised for fixing with 10 % buffered formalin. Tissue samples were stained with hematoxylin and eosin (H*&*E) and subsequently imaged using a digital microscope.

**1.8 Blood assay**

The hematology biocompatibility of TPAFN-F127 NPs was evaluated by complete blood counts (CBC). Briefly, TPAFN-F127 NPs (0.8 mg/kg, 4 mg/kg, and 20 mg/kg) were injected into the separate groups (8 mice per group) of mice via the tail vein. As a control, eight mice were injected with 200 μL PBS. After 1 week, about 1 mL whole blood collected from the eye socket of each mouse by a glass capillary was used for CBC. The analyzed items for TPAFN-F127 NPs including white blood cells, red blood cells, hemoglobin, packed cell volume, platelets, mean platelet volume, plateletcrit (PCT), mean corpuscular volume, mean corpuscular hemoglobin, and mean corpuscular hemoglobin concentration.

**1.9 *In vivo* imaging and *ex vivo* biodistribution analysis**

All *in vivo* fluorescent images were recorded on the IVIS Lumina II *In Vivo* Imaging System (PerkinElmer). A mouse xenograft tumor model was established through subcutaneous injection of 5×10^6^ HeLa cells into the right flank of mice. After the tumor volume reached about 200 mm^3^, the female BALB/c nude mice bearing subcutaneous HeLa tumor were given TPAFN-F127 NPs (200μL, 10 mg/kg) via tail vein injection. Then fluorescent images were captured by the Lumina II at various time points. At 24 h after injection of TPAFN-F127 NPs into HeLa-tumor bearing mice were sacrificed, then the major organs and tumor tissues were collected for the imaging study.

**1.10 Tissue preparation for LDI MS imaging**

Male Kunming mice injected with TPAFN-F127 NP (250 μL, 20 mg/kg) were sacrificed by cervical dislocation after 6 h, and organs studied in this paper were harvested within 10 min. Harvest organs were snap-frozen in liquid nitrogen and then stored in the fridge at −20°C. Then, tissue sections were thaw-mounted onto the conductive side of an indium tin oxide (ITO)–coated glass slide [Bruker Daltonics (Bremen, Germany)]. The ITO-coated glass slides with tissues were placed into a vacuum desiccator for about 30 min to completely dry the tissue.

**1.11 LDI MSI**

MSI was performed in positive reflection mode on an Ultraflextreme MALDI-TOF/TOF mass spectrometer [Bruker Daltonics (Billerica, MA)] equipped with a smart beam II Nd:YAG 355-nm laser. Each mass spectrometer was acquired by adding 200 laser shots with the laser operating at 2000 Hz. Spatial resolution for tissue section imaging was set as 50 to 100 mm, the spatial resolution of tissue homogenate imaging for quantification was set as 200 mm. Before imaging, the laser power energy was adjusted to the optimal energy to acquire the best fingerprint ion signal of TPAFN-F127 NPs. For quantification, the laser energy was kept constant for the whole process. The FlexImaging 4.0 software provided by Bruker Daltonics was used to profile the MSI results.

**1.12 Quantification of TPAFN-F127 NPs in organs by imaging mass spectrometry**

Quantification of TPAFN-F127 NPs in various organs was following previously reported imaging mass spectrometry method [1, 2]. The detailed protocol was introduced as below: The organs (heart, liver, lung, kidney, spleen) from normal mice were harvested and weighed to prepare tissue homogenate. The weighed organs were transferred into centrifuge tubes. For better lysis, the tissue with a homogenizer, 1% sodium dodecyl sulfate (SDS) solution was added to the weighed organs (5 μL/mg tissue) to lyses cells. The lysis homogenate was then heated at 70°C for 2 h to get a clear solution. Then the homogenates were spiked with a series of different concentrations of TPAFN-F127 NPs for each organ (20, 10, 5, 1, 0.5 μg/ml). The spiked tissue homogenate (about 200 μL) was added into a 1 mL disposable syringe (the end was cut open) and freezing in liquid nitrogen for 30 s for a cylindrical solid. Then the syringe with homogenate solid was placed in a -20 ºC freezer for at least half an hour. After that, the solid was pushed out of the syringe and the solid was sectioned into 25 μm thickness slices in a Leica CM1950 cryostat (Leica Biosystems, Nussloch, Germany). Three parallel slices for each concentration of TPAFN-F127 NPs homogenate were prepared. Then the slices were thaw-mounted onto the conductive side of an ITO-coated glass slide (Bruker Daltonics, Bremen, Germany) and dried for 30 min in a vacuum desiccator before mass spectrometry imaging analysis. After imaging, the average ion intensity of TPAFN-F127 NPs (choosing the strongest isotope peak *m/z* = 564.2 as representative) could be read out with FlexImaging 4.0 software (Bruker Daltonics) for each slice. Then plotted the average ion intensity to the concentrations of TPAFN-F127 NPs, then the calibration curve could be obtained. Calibration curves for the lung, spleen, and liver are shown in Figure S16.


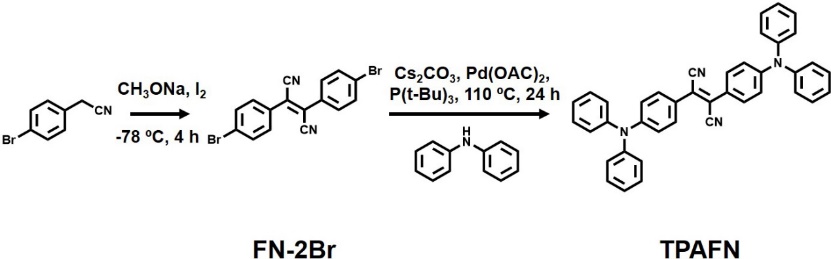


**Scheme S1**. The synthetic route of AIE molecule (TPAFN).


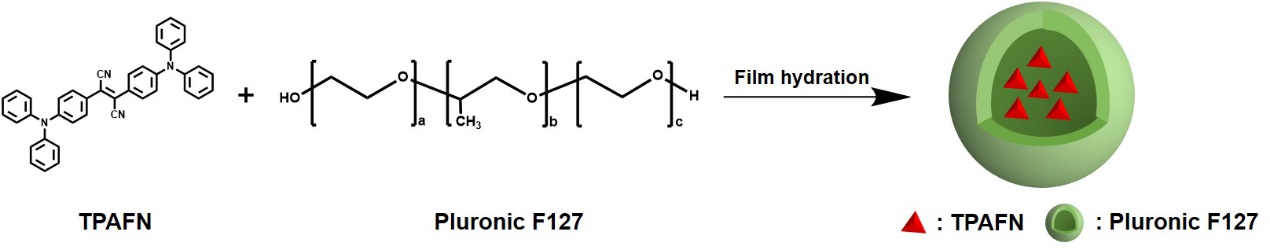


**Scheme S2**. The preparation of TPAFN-F127 NPs via a thin-film hydration method.


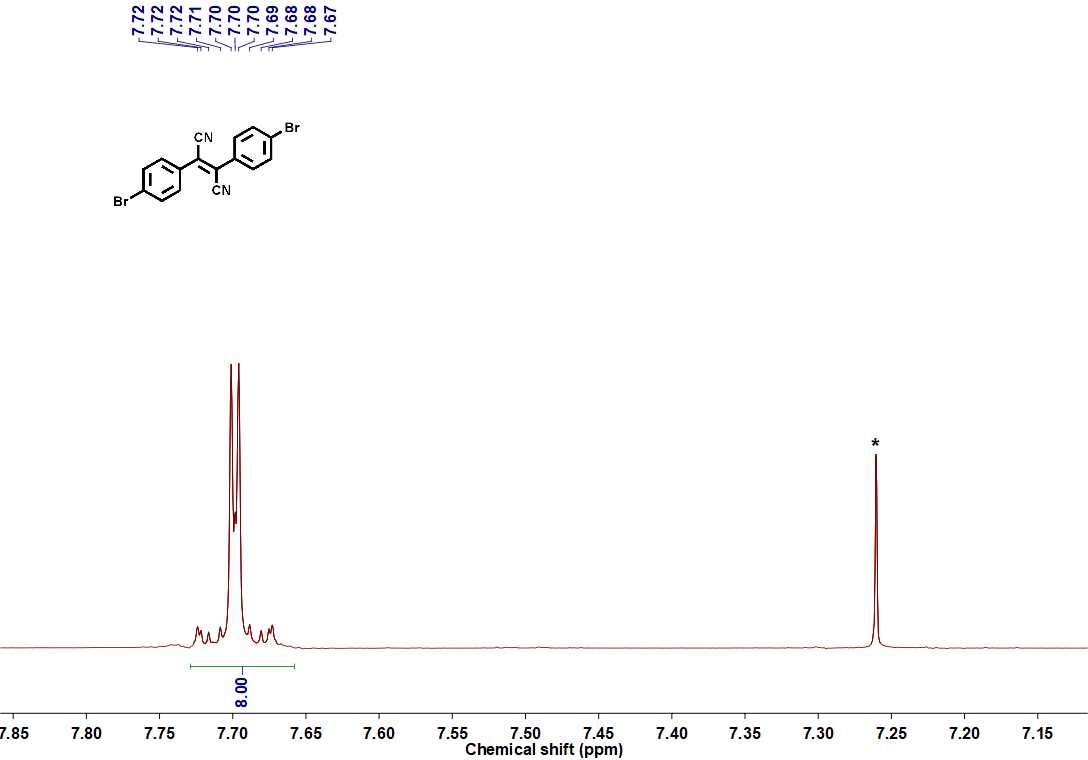


**Figure S1**. ^1^H NMR spectrum of FN-2Br.


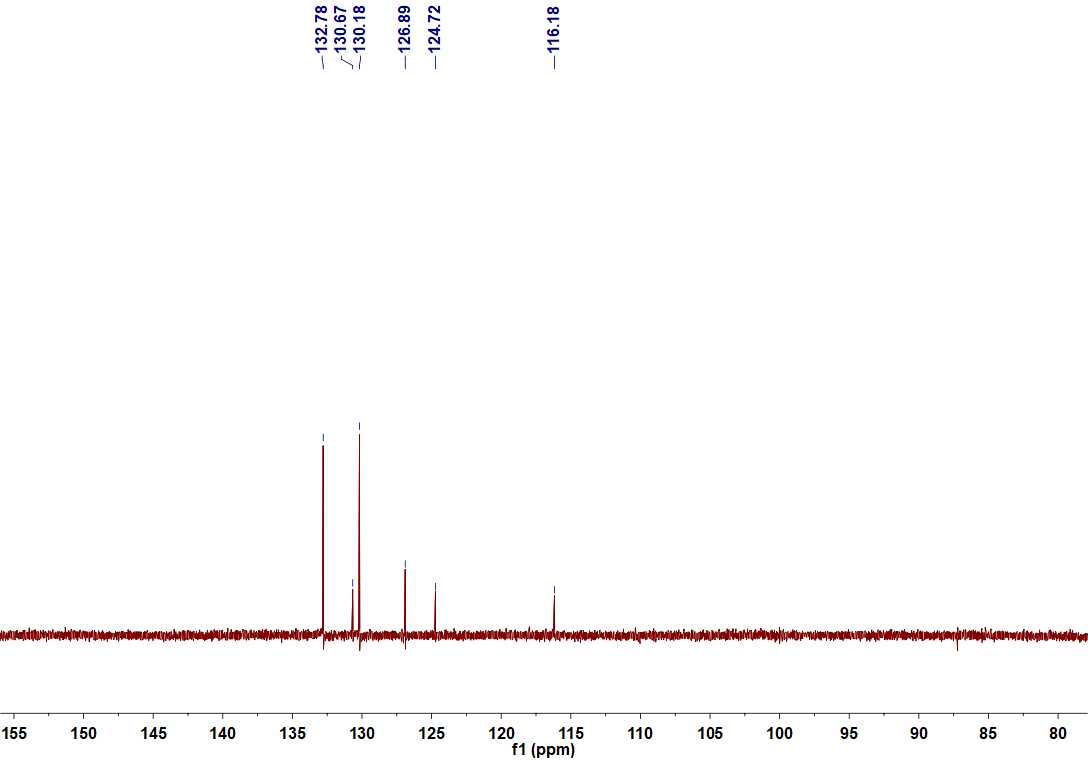


**Figure S2**. ^13^C NMR spectrum of FN-2Br.


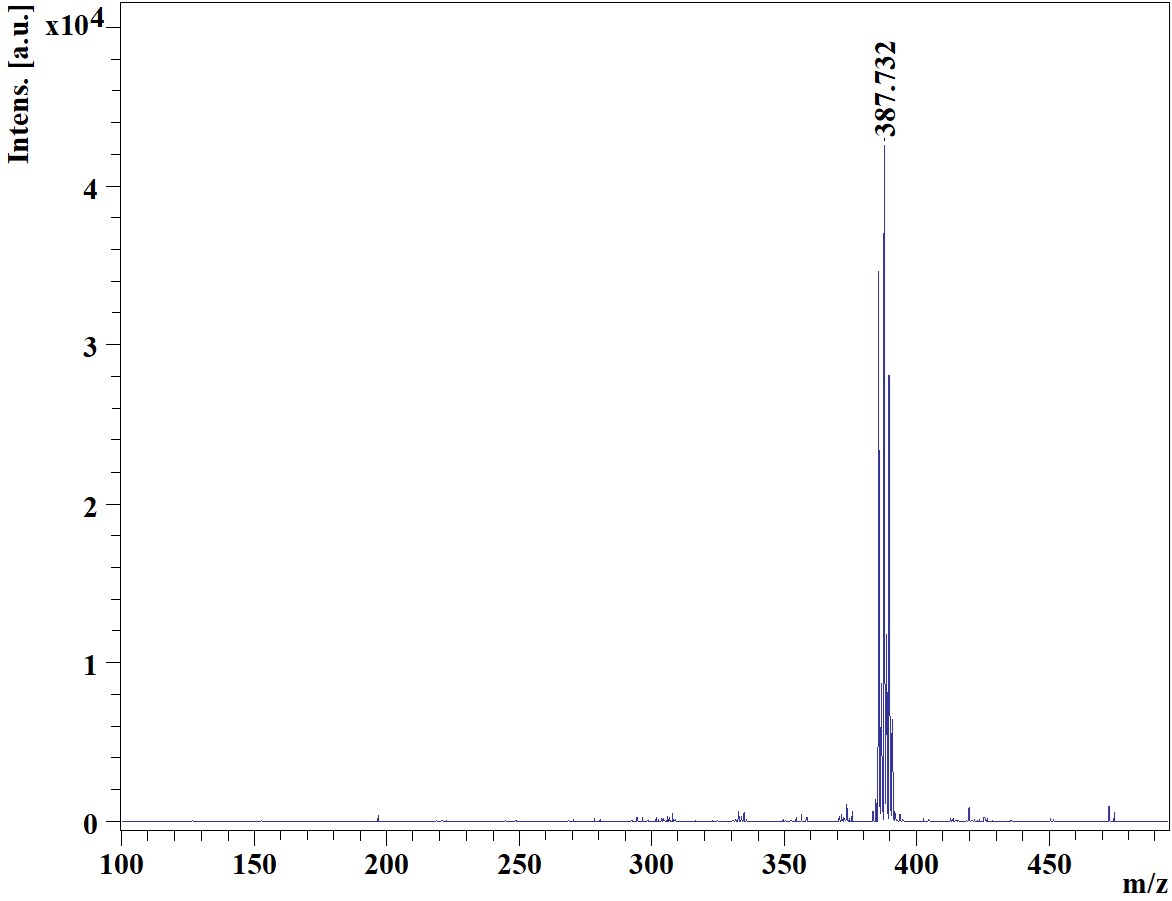


**Figure S3**. MALDI-TOF MS spectrum of FN-2Br.


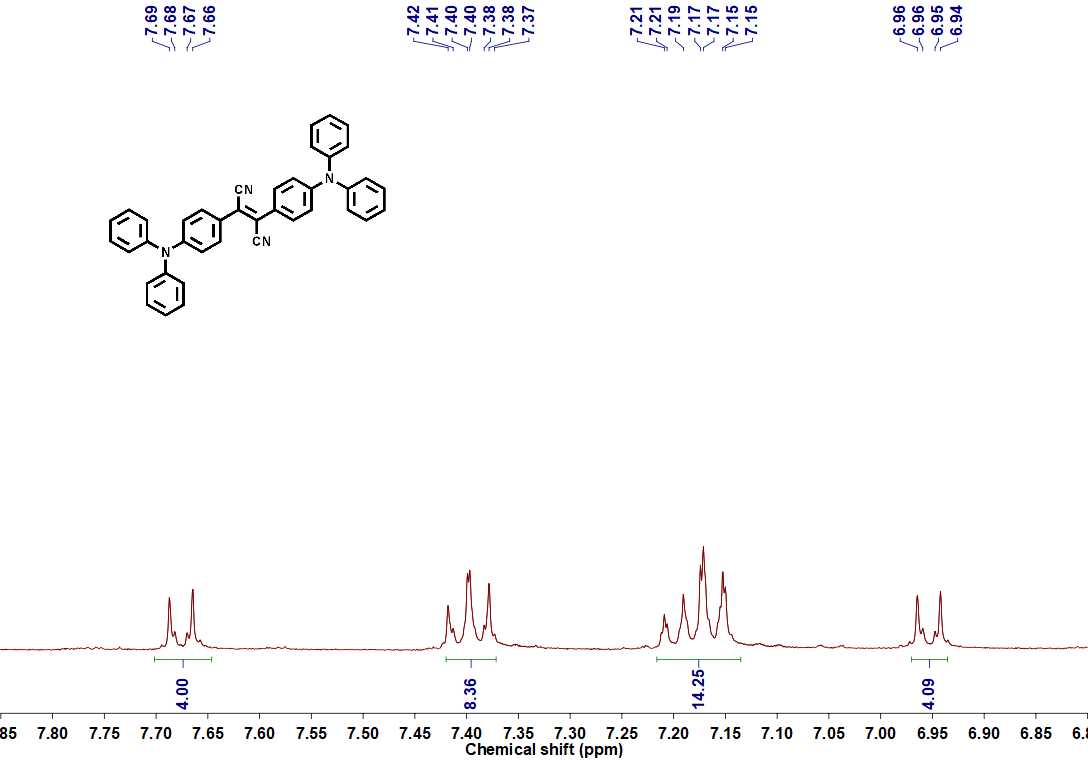


**Figure S4**. ^1^H NMR spectrum of TPAFN.


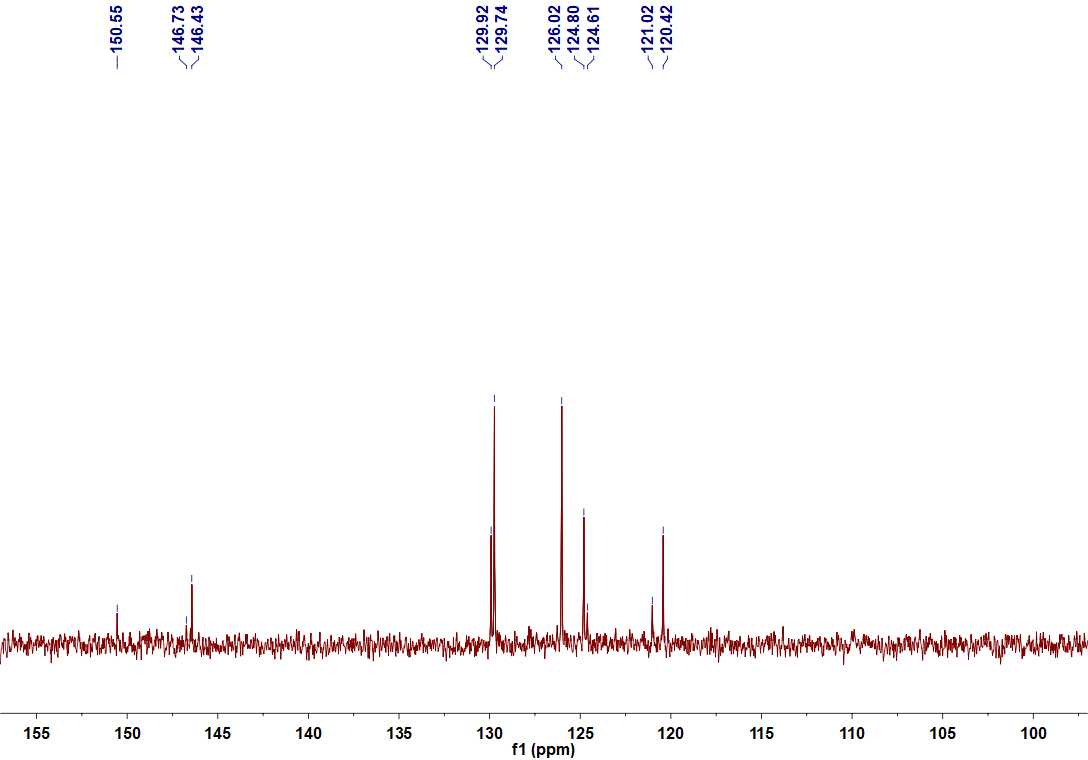


**Figure S5**. ^13^C NMR spectrum of TPAFN.


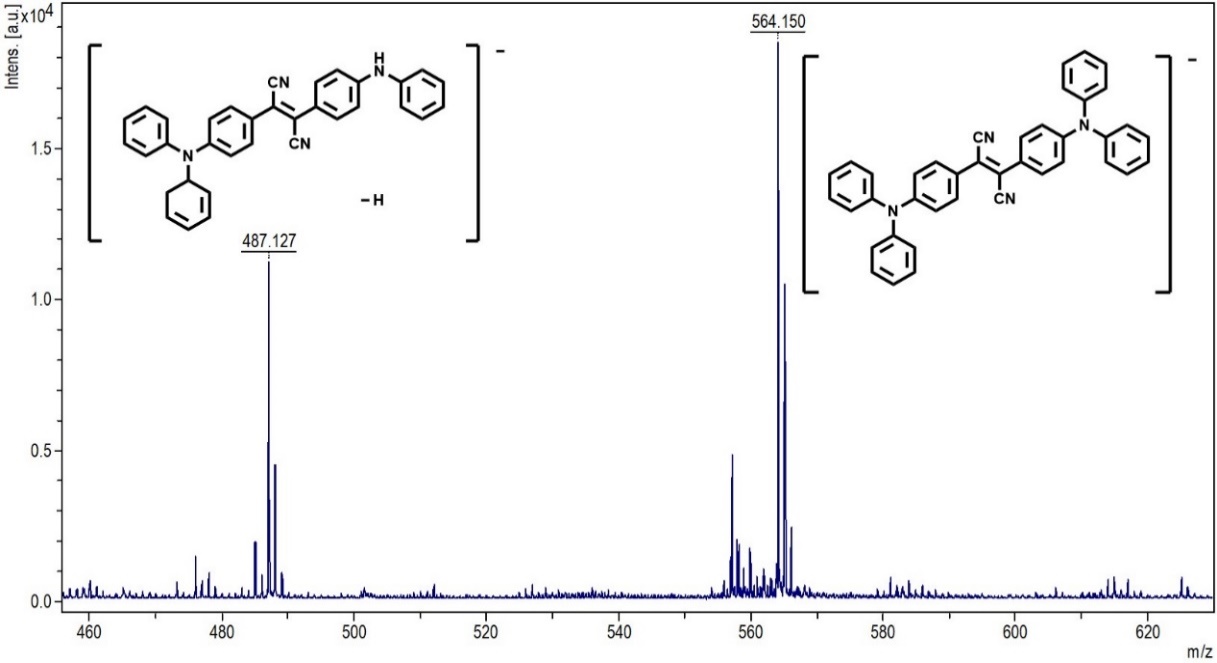


**Figure S6**. MALDI-TOF MS spectrum of TPAFN.


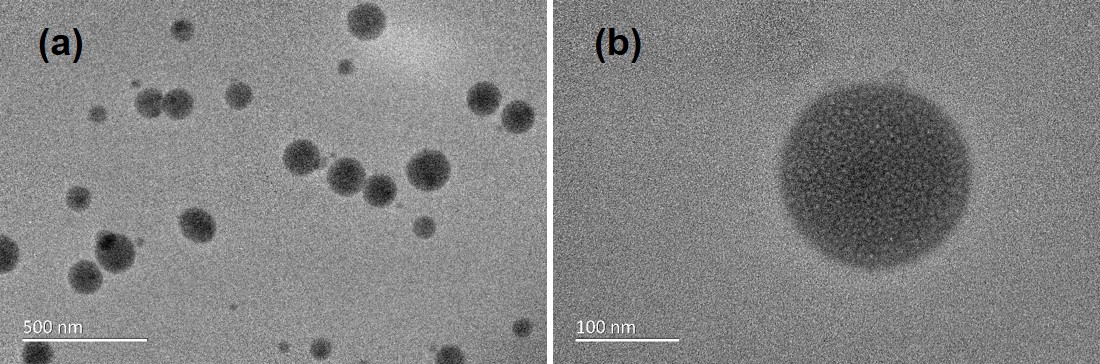


**Figure S7**. TEM image of TPAFN-F127 NPs.


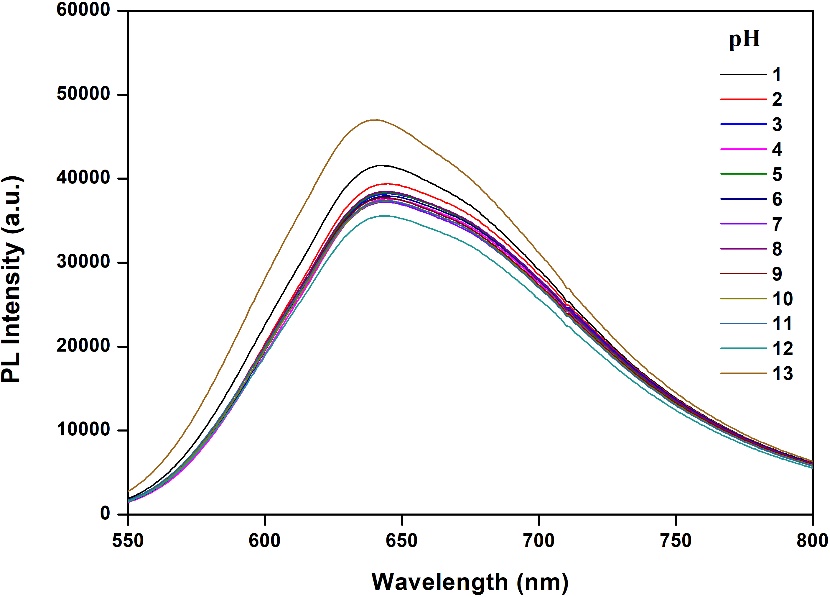


**Figure S8**. The variation of fluorescent intensity of TPAFN-F127 NPs incubated with different mediums with various pH buffers.

**Table S1**

Changes of hematology parameters of mice induced by TPAFN-F127 NPs (n = 8)

| Parameters | Value (mean ± SD) by exposure group | | | | | |
| --- | --- | --- | --- | --- | --- | --- |
|  | Control | PBS | 0.8 mg/kg | 4 mg/kg | 20 mg/kg |  |
| White blood cells (×10^3^/μL) | 3.42 ± 0.53 | 3.69 ± 0.52 | 3.64 ± 0.24 | 2.78 ± 0.63^*^ | 3.00 ± 0.55 |  |
| Red blood cells (×10^6^/μL) | 7.62 ± 0.41 | 8.15 ± 0.24 | 7.61 ± 0.71 | 7.91 ± 0.28 | 7.48 ± 0.39^**^ |  |
| Hemoglobin (g/dL) | 124.17 ± 8.09 | 128.17 ± 4.52 | 118.83 ± 5.84^*^ | 123.33 ± 3.20 | 111.00 ± 5.74^**^ |  |
| Packed cell volume (%) | 37.12 ± 1.88 | 41.30 ± 0.77 | 38.68 ± 2.98 | 41.33 ± 2.25 | 38.83 ± 2.04^*^ |  |
| Platelets (×10^3^/μL) | 964.67 ± 73.51 | 824.33 ± 130.46 | 716.00 ± 109.37 | 742.67 ± 137.25 | 962.67 ± 101.21 |  |
| Mean platelet volume (fL) | 6.13 ± 0.12 | 6.05 ± 0.20 | 6.15 ± 0.26 | 6.07 ± 0.07 | 6.10 ± 0.18 |  |
| PCT (%) | 0.59 ± 0.04 | 0.49 ± 0.08 | 0.40 ± 0.08 | 0.47 ± 0.08 | 0.58 ± 0.07 |  |
| Mean corpuscular volume (fL) | 48.85 ± 1.16 | 50.05 ± 1.11 | 51.72 ± 1.77 | 52.07 ± 0.80^**^ | 51.92 ± 1.00^*^ |  |
| Mean corpuscular hemoglobin (pg) | 16.12 ± 0.52 | 15.71 ± 0.23 | 16.18± 1.19 | 15.38 ± 0.26 | 15.00 ± 0.45^**^ |  |
| Mean corpuscular Hemoglobin concentration (g/dL) | 329.00 ± 4.76 | 318.83 ± 5.18 | 308.17 ± 12.99 | 296.33 ± 7.56^**^ | 288.83 ± 3.67^**^ |  |

^*^ A value significantly (P < 0.05) differed from the PBS group.

^**^ A value significantly (P < 0.01) differed from the PBS group.


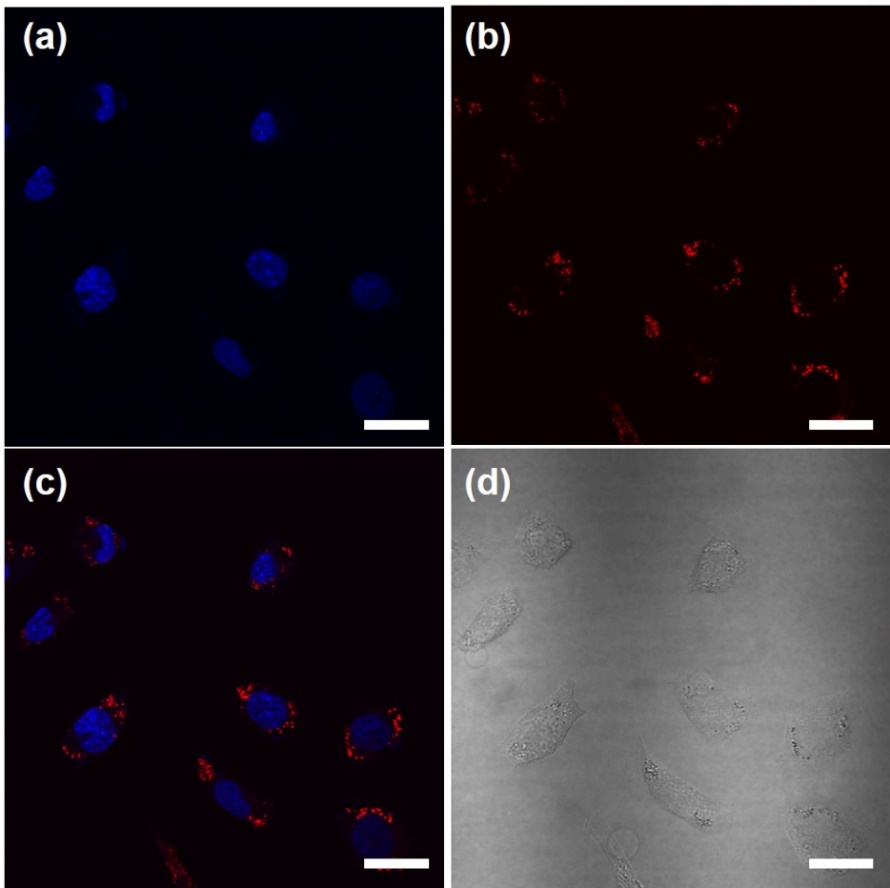


**Figure S9**. CLSM imaging of L929 cells after incubation with TPAFN-F127 NPs for 3 h at 37 ºC. The concentration of TPAFN-F127 NPs is 5 μg mL^–1^. Scale bar: 20 μm.


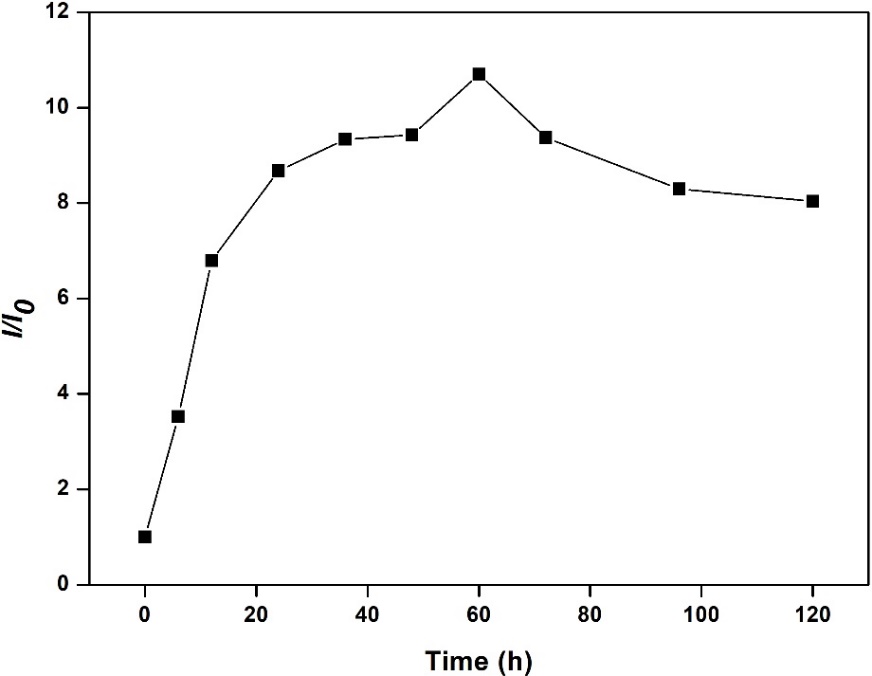


**Figure S10**. Variation in the intensity at different time points after tail-vein injection of TPAFN-F127 NPs. *I*_0_ refers to the intensity of the tumor region in the control group. The signal intensity of the tumor tissue was recorded on the regions enclosed by the red circles in Figure 4e, respectively.


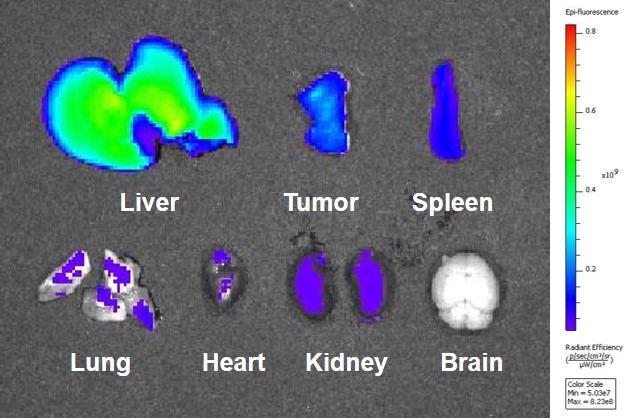


**Figure S11**. *Ex vivo* fluorescence imaging of Tumor and major organs after 24 h post intravenous injection of TPAFN-F127 NPs (200 μL, 10 mg/kg).





**Figure S12**. Histogram of the PL intensity of the organs (heart, liver, spleen, lung, and kidney), brain, and tumor.


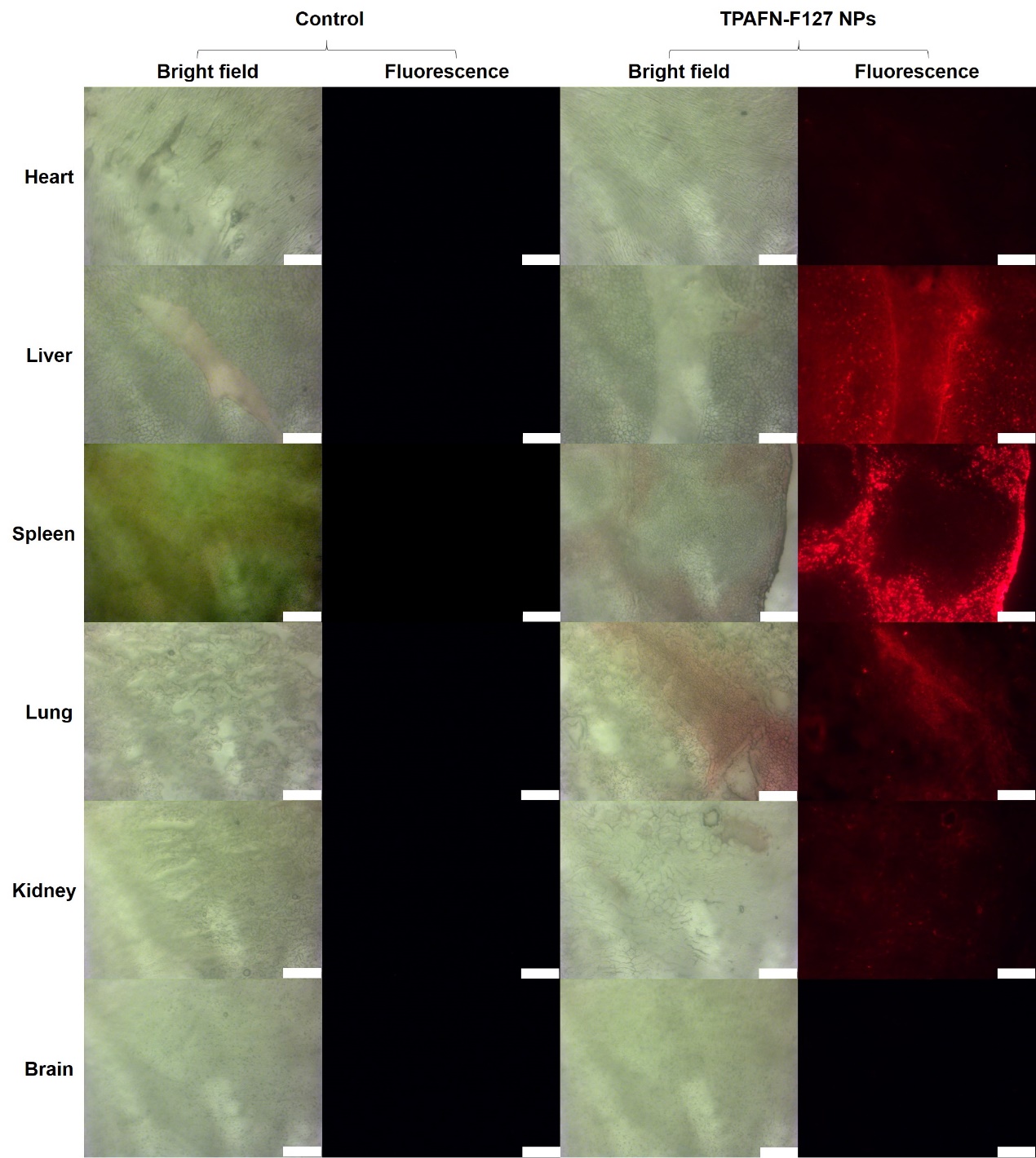


**Figure S13**. Representative *ex vivo* fluorescence imaging of various types of tissue slices from mice treated with TPAFN-F127 NPs (200 μL, 10 mg/kg) after 24 h post-injection; scale bar: 200 μm.


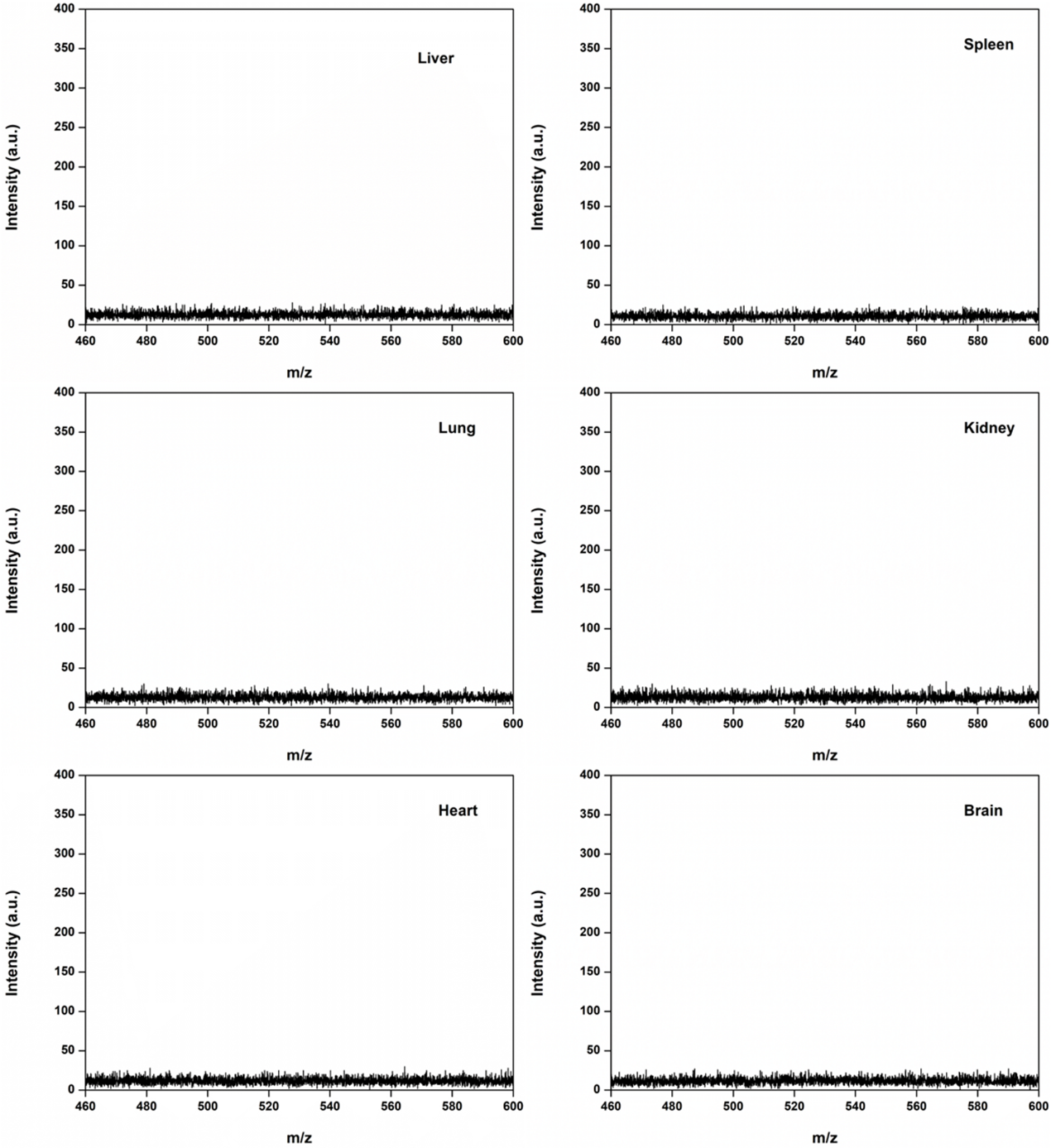


**Figure S14**. Representative LDI mass spectra of normal mice tissue slice of liver, spleen, lung, kidney, heart, and brain in negative ion mode.


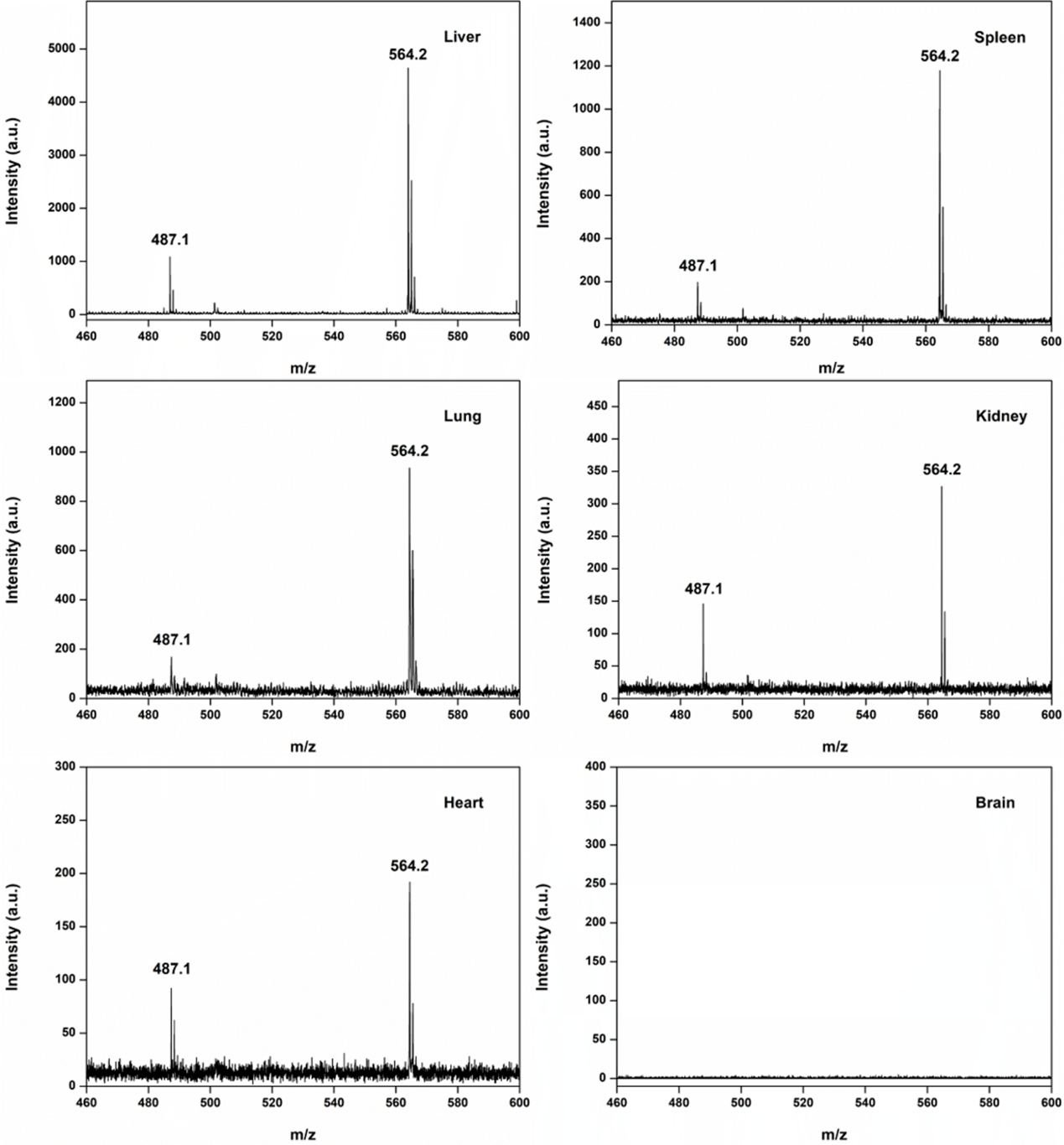


**Figure S15**. Representative LDI mass spectra of TPAFN-F127 NPs-injected normal mice tissue slice of liver, spleen, lung, kidney, heart, and brain in negative ion mode.


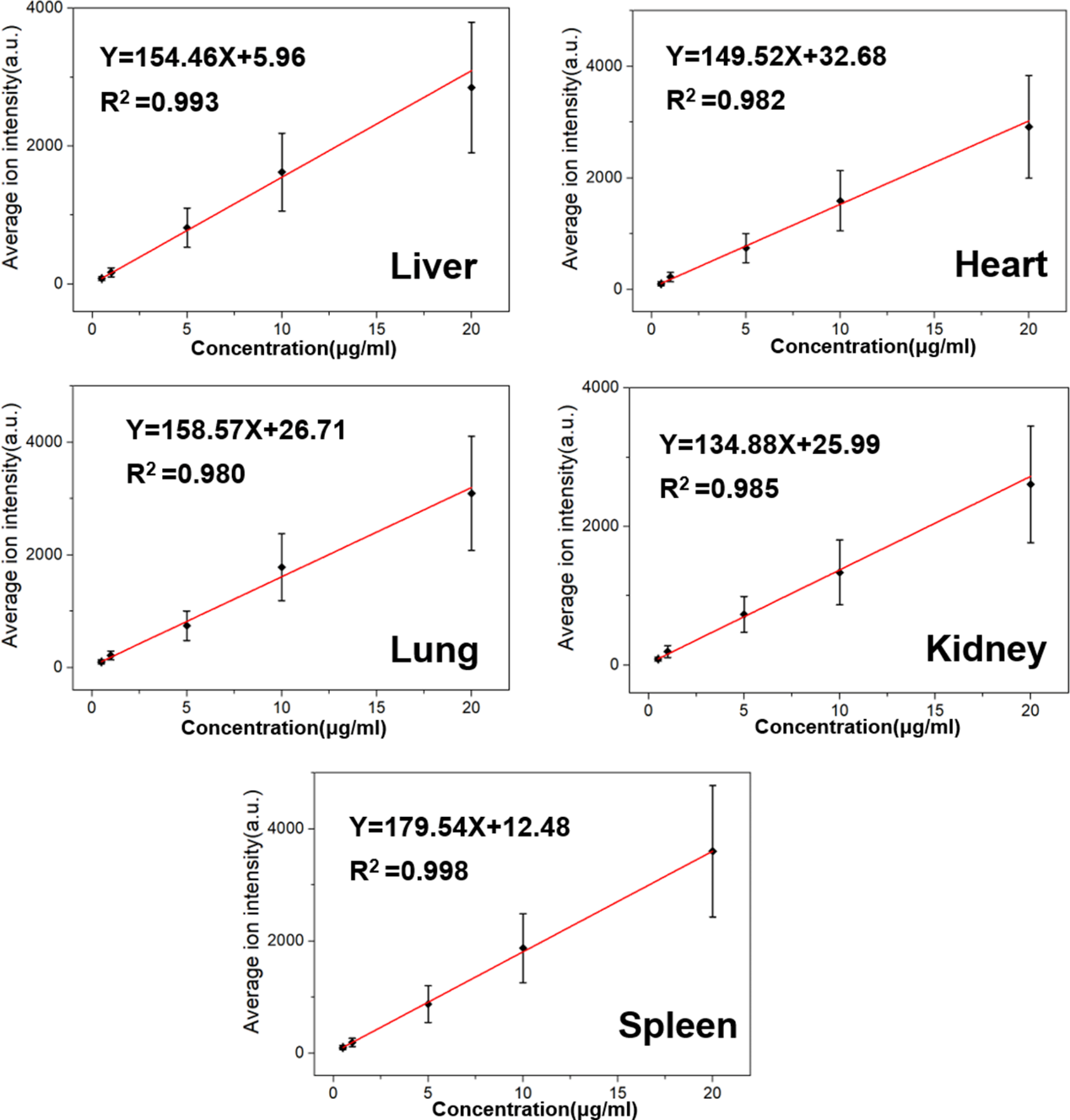


**Figure S16**. Standard calibration curves for TPAFN-F127 NPs in various organs including liver, heart, lung, kidney, and spleen.


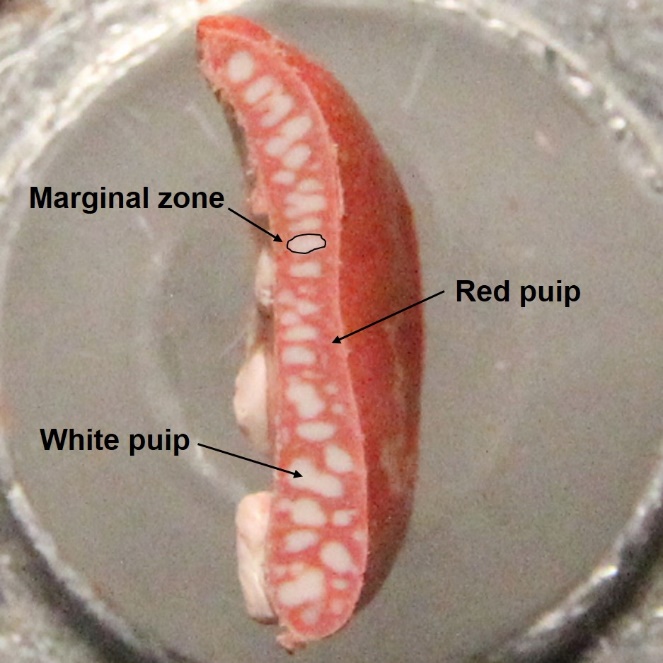


**Figure S17**. Photograph of splenic tissues.


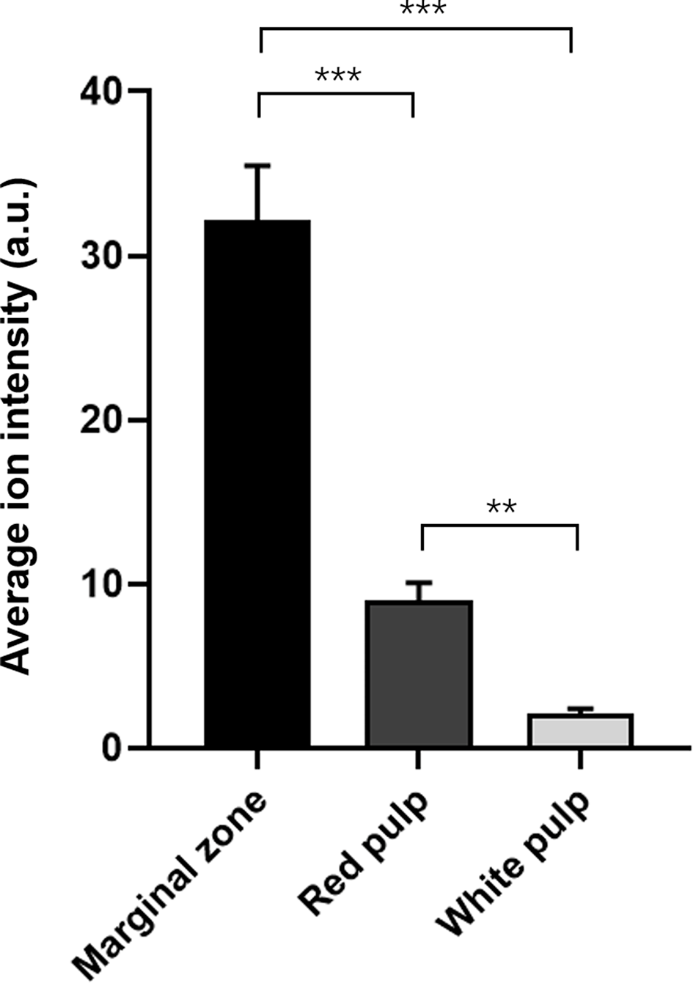


**Figure S18**. Quantitative results of TPAFN-F127 NPs in the white pulp, red pulp, and marginal zones in the spleen tissue section. Ten regions of interest in the LDI MSI image were selected for white pulp, red pulp, and marginal zone, respectively. Each region contains about 100-pixel points. ***P < 0.001.

**References**

[1] S. Chen, C. Xiong, H. Liu, et al. "Mass Spectrometry Imaging Reveals the Sub-Organ Distribution of Carbon Nanomaterials," Nature Nanotechnology, vol. 10, no. 2, pp. 176, 2015.

[2] J. Xue, H. Liu, S. Chen, et al. "Mass Spectrometry Imaging of the in Situ Drug Release from Nanocarriers," Science Advances, vol. 4, no. 10, pp. eaat9039, 2018.
